# A small-molecule degrader of TET3 as treatment for anorexia nervosa in an animal model

**DOI:** 10.1073/pnas.2300015120

**Published:** 2023-04-10

**Authors:** Haining Lv, Jonatas Catarino, Da Li, Beibei Liu, Xiao-Bing Gao, Tamas L. Horvath, Yingqun Huang

**Affiliations:** ^a^Department of Obstetrics, Gynecology and Reproductive Sciences, Yale University School of Medicine, New Haven, CT 06520; ^b^Department of Obstetrics and Gynecology and Center for Reproductive Medicine, Affiliated Drum Tower Hospital, Medical School of Nanjing University, Nanjing 210008, China; ^c^Department of Comparative Medicine, Yale University School of Medicine, New Haven, CT 06520; ^d^Center of Reproductive Medicine, National Health Commission Key Laboratory of Reproductive and Genetic Medicine, Shengjing Hospital of China Medical University, Shenyang 110004, China; ^e^Yale Center for Molecular and Systems Metabolism, Yale University School of Medicine, New Haven, CT 06520; ^f^Department of Neuroscience, Yale University School of Medicine, New Haven, CT 06520

**Keywords:** AgRP, feeding, anxiety, depression

## Abstract

Anorexia nervosa (AN) is a psychiatric illness with the highest mortality. Current treatment options have been limited to psychotherapy and nutritional support, with low efficacy and high relapse rates. Hypothalamic AgRP (agouti-related peptide) neurons that coexpress AGRP and neuropeptide Y (NPY) play a critical role in driving feeding while also modulating other complex behaviors. We have previously reported that genetic ablation of *Tet3*, which encodes a member of the TET family dioxygenases, specifically in AgRP neurons in mice, activates these neurons and increases the expression of AGRP, NPY, and the vesicular GABA transporter (VGAT), leading to hyperphagia and anxiolytic effects. Bobcat339 is a synthetic small molecule predicted to bind to the catalytic pockets of TET proteins. Here, we report that Bobcat339 is effective in mitigating AN and anxiety/depressive-like behaviors using a well-established mouse model of activity-based anorexia (ABA). We show that treating mice with Bobcat339 decreases TET3 expression in AgRP neurons and activates these neurons leading to increased feeding, decreased compulsive running, and diminished lethality in the ABA model. Mechanistically, Bobcat339 induces TET3 protein degradation while simultaneously stimulating the expression of AGRP, NPY, and VGAT in a TET3-dependent manner both in mouse and human neuronal cells, demonstrating a conserved, previously unsuspected mode of action of Bobcat339. Our findings suggest that Bobcat339 may potentially be a therapeutic for anorexia nervosa and stress-related disorders.

## Significance

Anorexia nervosa is a life-threatening psychiatric illness with poorly understood cellular and molecular mechanisms and limited treatment options. Emerging evidence points to the importance of hypothalamic agouti-related peptide (AgRP) neurons in anorexia nervosa, but the underlying molecular mechanisms linking AgRP neurons to this illness have remained ill-defined. Here, we show that Bobcat339, a synthetic small molecule that controls TET3 in AgRP neurons, is able to mitigate anorexia nervosa and associated anxiety/depressive behaviors in a murine model. We show that Bobcat339 acts to destabilize TET3 protein in AgRP neurons and that this regulation is conserved in human and mouse cells. We propose that Bobcat339 should be pursued as a therapeutic for anorexia nervosa and perhaps cancer-induced anorexia and associated mood disorders.

Anorexia nervosa (AN) is a severe eating disorder characterized by low caloric intake, intense fear of gaining weight, and excessive physical activity. Individuals with AN frequently exhibit stress symptoms including anxiety, depression, and obsessive–compulsive disorders (1–3). Females are nine times more often affected by AN than males; the highest incidence rate of AN in females is during adolescence (1, 4). Despite AN being a psychiatric illness with the highest mortality (1, 4), current treatment options have been limited to psychotherapy and nutritional support, with low efficacy and high relapse rates.

The agouti-related peptide (AGRP)-expressing neurons that coexpress neuropeptide Y (NPY) reside in the arcuate nucleus (ARC) of the hypothalamus. These AgRP neurons play a central role in driving feeding while also modulating other complex behaviors (5–13). Energy deprivation (e.g., food restriction) activates these neurons leading to increased production of AGRP, NPY, and gamma-aminobutyric acid (GABA), which act in concert to drive food intake and decrease energy expenditure (5). AGRP, NPY, and GABA can also elicit anxiolytic effects, consistent with the AgRP circuitry in modulation of anxiety, depression, and obsessive–compulsive behaviors (14, 15). The ten-eleven translocation (TET) family of proteins, composed of TET1, TET2, and TET3, initiate DNA demethylation by oxidizing 5-methylcytosine to 5-hydroxymethylcytosine (16, 17). Recently, we have uncovered TET3 as a critical regulator of feeding and stress response through AgRP neurons (18). We showed that CRISPR-mediated TET3 knockdown specifically in AgRP neurons in mice induces hyperphagia and reduces stress-like behaviors. Mechanistically, TET3 deficiency activates AgRP neurons and increases the expression of AGRP, NPY, and the vesicular GABA transporter (VGAT, encoded by *SLC32A1*) and that this regulation of gene expression by TET3 occurs both in mouse models and human cells (18). In the current work, we used a well-established mouse model of activity-based anorexia (ABA) (2, 3) to demonstrate that a small-molecule degrader of TET3 is able to effectively attenuate AN and anxiety/depressive-like behaviors, at least in part, via decreasing TET3 expression in AgRP neurons.

## Results

### Bc Destabilizes TET3 Protein in Neuronal Cells.

**Fig. 1.**
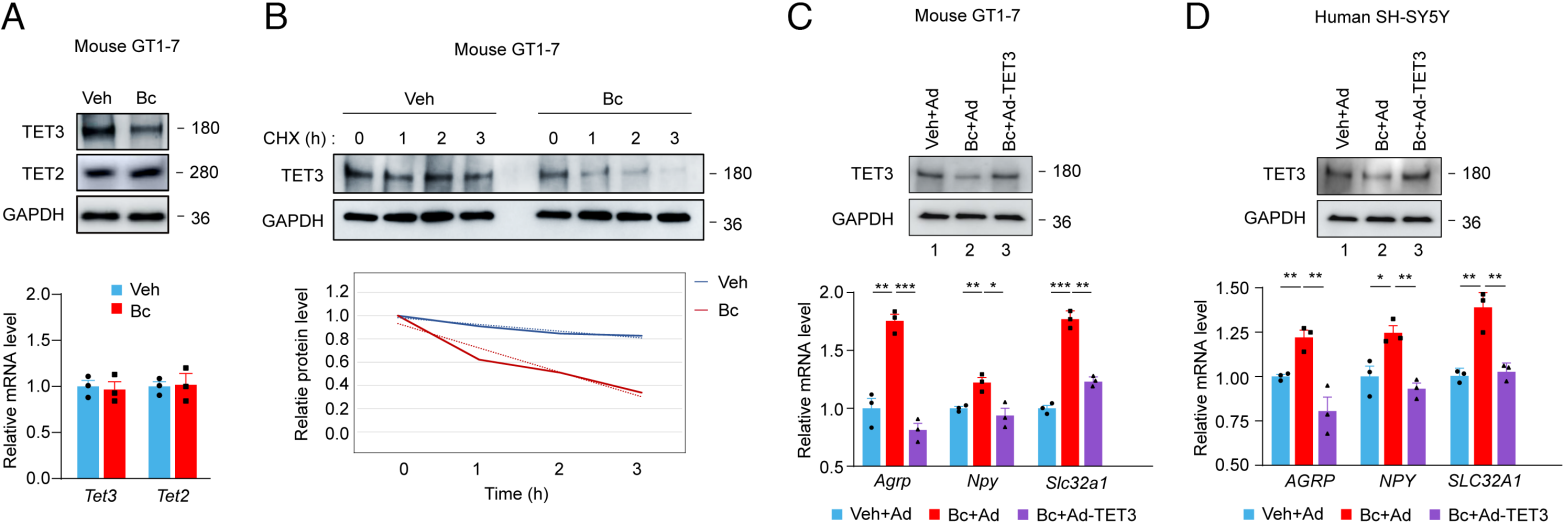
Bc induces TET3 protein degradation and increases the expression of *Agrp*/*AGR*P, *Npy*/*NPY*, and *Slc32a1*/*SLC32A1* in a TET3-dependent manner. (*A*) Mouse GT1-7 cells were incubated with vehicle or Bc at a final concentration of 10 μM in growth media for 6 h, followed by RNA and protein extraction and analyses. *Top*, representative immunoblots for TET3 and TET2 with glyceraldehyde-3-phosphate dehydrogenase (GAPDH) as a loading control showing decreased TET3 (but not TET2) protein in Bc-treated cells. *Bottom*, qPCR of *Tet3* and *Tet2* showing no significant difference between the groups. *n* = 3 per group in technical replicates, 2-tailed Student’s *t* test. (*B*) GT1-7 cells were incubated with vehicle or Bc at a final concentration of 10 μM for 3 h, followed by time course analysis of TET3 in the presence of cycloheximide (CHX) at a final concentration of 50 μg/mL. Cells were harvested at 0, 1, 2, and 3 h after addition of CHX. Bc was present in the growth media for a total of 6 h. (*C* and *D*) GT1-7 or SH-SY5Y cells were incubated with vehicle plus GFP-expressing adenovirus (Veh+Ad), Bc at 10 μM plus Ad (Bc+Ad), or Bc at 10 μM plus TET3-expressing adenovirus (Bc+Ad-TET3). Protein and RNA were isolated 48 h later and analyzed. *Top*, representative immunoblots for TET3 with GAPDH as a loading control. *Bottom*, qPCR of *Agrp*/*AGRP*, *Npy*/*NPY*, *and Slc32a1/SLC32A1*. *n* = 3 per group in technical replicates, 1-way ANOVA with Tukey’s posttest. All data represent mean ± SEM. *P < 0.05, **P < 0.01, and ***P < 0.001. All data are representative of at least two independent experiments.

The catalytic domain of the three TET enzymes is highly conserved, although each of the members exhibits varying substrate preferences and catalytic activity (19). Bobcat339 (herein called Bc) is a synthetic cytosine derivative initially reported to inhibit the enzymatic activity of TET1 and TET2, but its effects in vivo and on TET3 were not defined (20). Recently, it was found that Bc on its own had a negligible inhibitory activity against TET1 and TET2 in the absence of contaminating copper(II) (21). Nonetheless, as Bc was predicted to bind to the catalytic sites of all three TET enzymes based on the crystal structure of TET2–DNA complex (20), we reasoned that Bc might affect TET3. Thus, we incubated GT1-7, an immortalized mature mouse hypothalamic GnRH neuronal cell line, with Bc at 10 μM for 6 h and unexpectedly found a decrease in the protein level of TET3 (Fig. 1 *A*, *Top*) without affecting its messenger ribonucleic acid (mRNA) abundance (Fig. 1 *A*, *Bottom*). Bc did not affect TET2 expression (Fig. 1*A*). No expression of TET1 was detected in GT1-7 cells, consistent with a previous study reporting negligible TET1 expression in the adult mouse hypothalamus (18). It is not unprecedented that compounds initially developed as protein function modulators are later serendipitously found to promote protein degradation (22, 23). To test whether Bc might affect TET3 protein stability, we performed time course experiments in the presence of cycloheximide (CHX), a protein synthesis inhibitor. We found that TET3 was less stable in Bc-treated vs. vehicle-treated cells (Fig. 1 *B*, *Upper*). While TET3 remained stable in vehicle-treated cells, it became dramatically unstable in Bc-treated cells with a half-life of ~2 h (Fig. 1 *B*, *Bottom*). Since we used Bobcat339 purchased from Sigma-Aldrich shown to be free from Cu(II) contamination (21), we conclude that Bc induces TET3 protein degradation likely without affecting its enzymatic activity, a discovery not previously documented.

### Bc Affects the Expression of *Agrp/AGRP*, *Npy/NPY*, and *Slc32a1/SLC32A1*.

Given that TET3 inhibits the expression of *Agrp*/*AGRP*, *Npy*/*NPY*, and *Slc32a1*/*SLC32A1* (18) and that Bc destabilizes TET3 protein (Fig. 1 *A* and *B*), we tested whether exposing cells to Bc would increase the expression of *Agrp*/*AGRP*, *Npy*/*NPY*, and *Slc32a1*/*SLC32A1* in a TET3-dependent manner. Thus, GT1-7 cells were incubated with Bc in the presence or absence of exogenous TET3 expression from an adenoviral vector (Ad-TET3). While Bc expectedly decreased the level of TET3 protein (Fig. 1 *C*, *Top*, compare lane 2 to lane 1), exogenous TET3 expression restored it to the level of control (*Top*, compare lane 3 to lane 1). When mRNAs were examined, we observed increases in the mRNA levels of *Agrp*, *Npy*, and *Slc32a1* (Fig. 1 *C*, *Bottom*, compare red bars to blue bars) which were not seen when the level of TET3 protein was restored by exogenously expressed TET3 (compare purple bars to blue bars). Similar observations were made in SH-SY5Y human neuronal cells (Fig. 1*D*). Taken together, our results show that Bc stimulates the expression of *Agrp*/*AGRP*, *Npy*/*NPY*, and *Slc32a1*/*SLC32A1* in a TET3-dependent manner and that this regulation appears to be conserved in human and mouse cells.

### Bc Down-Regulates TET3 Protein in AgRP Neurons.

**Fig. 2.**
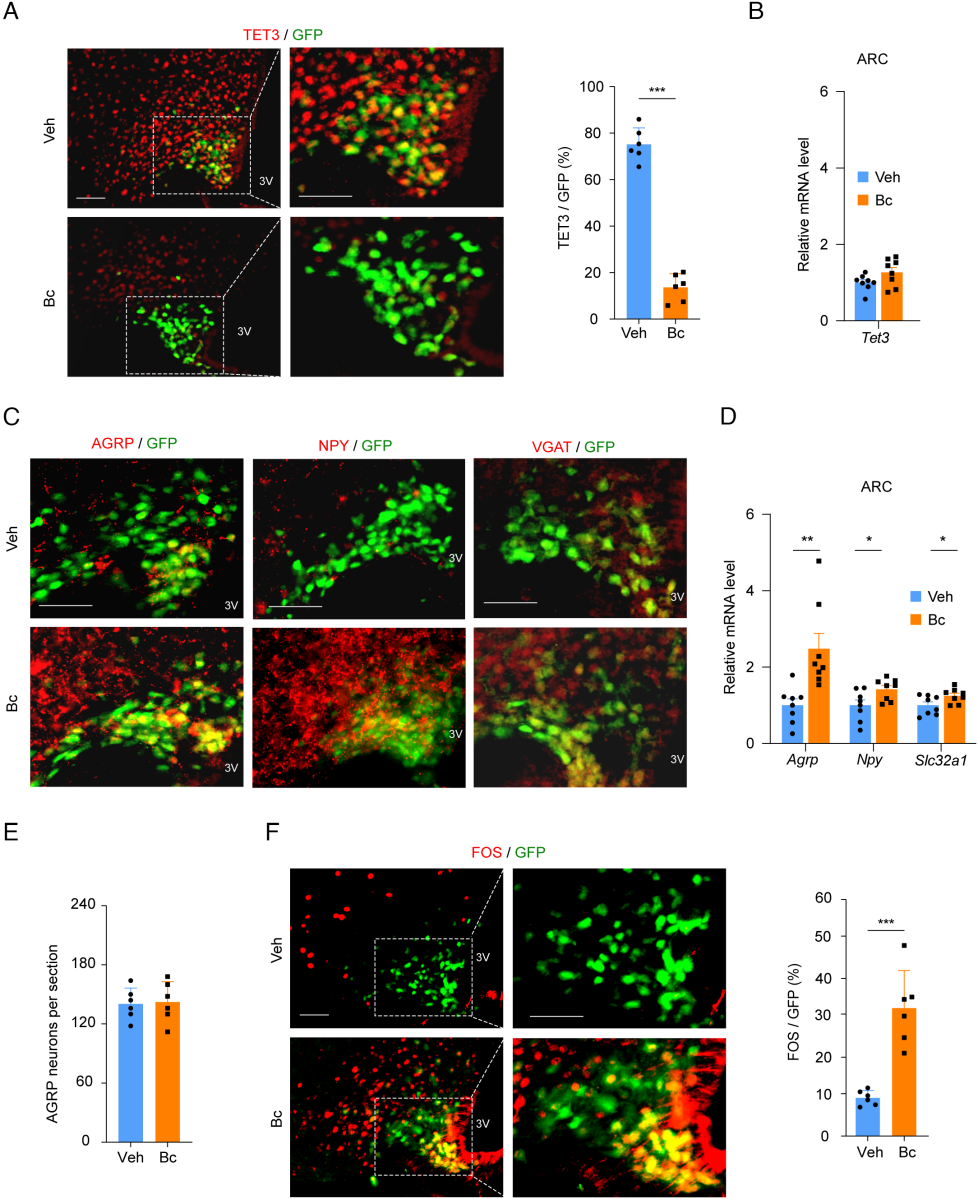
Effects of Bc on gene expression in the ARC. (*A*) Representative microphotographs and corresponding statistical analysis of TET3^+^ (red) AgRP neurons (green) showing decreased TET3 protein in AgRP neurons in Bc-treated mice. *n* = 6 animals per group. Each dot represents an animal. (*B*) qPCR of *Tet3* showing no significant change in the ARCs of Bc- vs. vehicle-treated animals. *n* = 8 animals per group. Each dot represents an animal. (*C*) Representative microphotographs of AGRP (red), NPY (red), and VGAT (red) showing a marked increase in their expressions in Bc-treated mice. (*D*) qPCR of indicated genes showing increased expressions of *Agrp, Npy*, and *Slc32a1* in the ARCs of Bc-treated animals. *n* = 8 animals per group. Each dot represents an animal. (*E*) Quantification of AgRP neurons in the ARCs of mice treated with vehicle or Bc showing no significant difference between the groups. *n* = 6 animals per group. Each dot represents an animal. (*F*) Representative microphotographs and corresponding statistical analysis of FOS^+^ (red) AgRP neurons (green) showing increased expression of FOS in AgRP neurons in Bc-treated mice. *n* = 6 animals per group. Each dot represents an animal. All data represent mean ± SEM. *P < 0.05, **P < 0.01, and ***P < 0.001 by two-tailed Student’s *t* test. 3V, third ventricle. (Scale bars, 50 μm.)

As exposing mice to Bc in drinking water induces hyperphagia, phenocopying CRISPR-mediated genetic TET3 knockdown in AgRP neurons (18), we suspected that Bc might inhibit TET3 expression in AgRP neurons. Thus, *Agrp-IRES-Cre::LSL-Cas9-GFP* mice (18) with GFP expression specifically in AgRP neurons were intraperitoneally (i.p.) injected with vehicle or Bc at 2.5 mg/kg body weight, followed by isolation of ARCs under fed conditions (9:00 to 11:00) 2 d later. This dose was chosen based on our dose–response studies (*SI Appendix*, Fig. S1*A*). TET2 expression was widespread (*SI Appendix*, Fig. S2, *Upper*) but was negligible in AgRP neurons (*SI Appendix*, Fig. S2, *Bottom*). Importantly, Bc did not significantly affect TET2 expression (*SI Appendix*, Fig. S2, *Upper*, compare *Left* to *Right*), consistent with in vitro findings (Fig. 1*A*). In contrast, TET3 was readily detected both in AgRP and non-AgRP cells, with a clear decrease in the number of TET3-positive AgRP neurons in Bc-treated animals (Fig. 2*A*), demonstrating decreased expression of TET3 protein in AgRP neurons. Given that Bc does not alter the mRNA abundance of *Tet3* in the ARC (Fig. 2*B*) and that Bc destabilizes TET3 protein in vitro (Fig. 1 *A* and *B*), we suggest that Bc destabilizes TET3 protein in AgRP neurons.

Notably, Bc treatment also decreased TET3 protein in non-AgRP cells (Fig. 2 *A*, *Left*), but the decrease appeared to be limited to the tip regions of the ARC even at a higher dose (4 mg/kg) (*SI Appendix*, Fig. S1*B*). The ARC neurons reside in the areas where the blood–brain barrier is modified to be more permeable to facilitate the access of blood-borne nutrients, hormones, and metabolites (24). We propose minimizing off-target effects of Bc through dose optimization (Discussion).

### Bc Up-Regulates the Expression of AGRP, NPY, and VGAT in the ARC.

Next, we detected increased productions of AGRP, NPY, and VGAT in Bc-exposed animals (Fig. 2*C*). Note, the GFP construct used for tagging AgRP neurons labels almost exclusively the perikaryon. The presence of AGRP, NPY, and VGAT outside the perikarya reflected the fact that these fast-firing neurons transport their neuromodulatory products rapidly to the neuronal processes. Increases in the mRNA levels of *Agrp*, *Npy*, and *Slc32a1* were also observed in the ARCs of animals treated with Bc (Fig. 2*D*). Furthermore, Bc treatment did not affect AgRP neuronal viability (Fig. 2*E*). These results show that Bc increases the expression of AGRP, NPY, and VGAT, likely through inhibiting TET3 protein expression in AgRP neurons, consistent with our in vitro findings (Fig. 1 *C* and *D*).

### Bc Increases the Number of FOS-Positive AgRP Neurons.

As genetic TET3 knockdown specifically in AgRP neurons activates these neurons (18), we tested whether Bc treatment might activate AgRP neurons using FOS (a marker for neuronal activation) as a readout. Thus, ARCs were isolated under fed conditions (9:00 to 11:00) from mice treated with Bc or vehicle as above (Fig. 2 *A*–*D*). Immunofluorescence analysis revealed increased FOS expression in AgRP neurons in Bc- vs. vehicle-treated mice (Fig. 2*F*). These results suggest that Bc down-regulates TET3 protein in AgRP neurons, leading to neuronal activation.

### Bc Mitigates Anorexia.

**Fig. 3.**
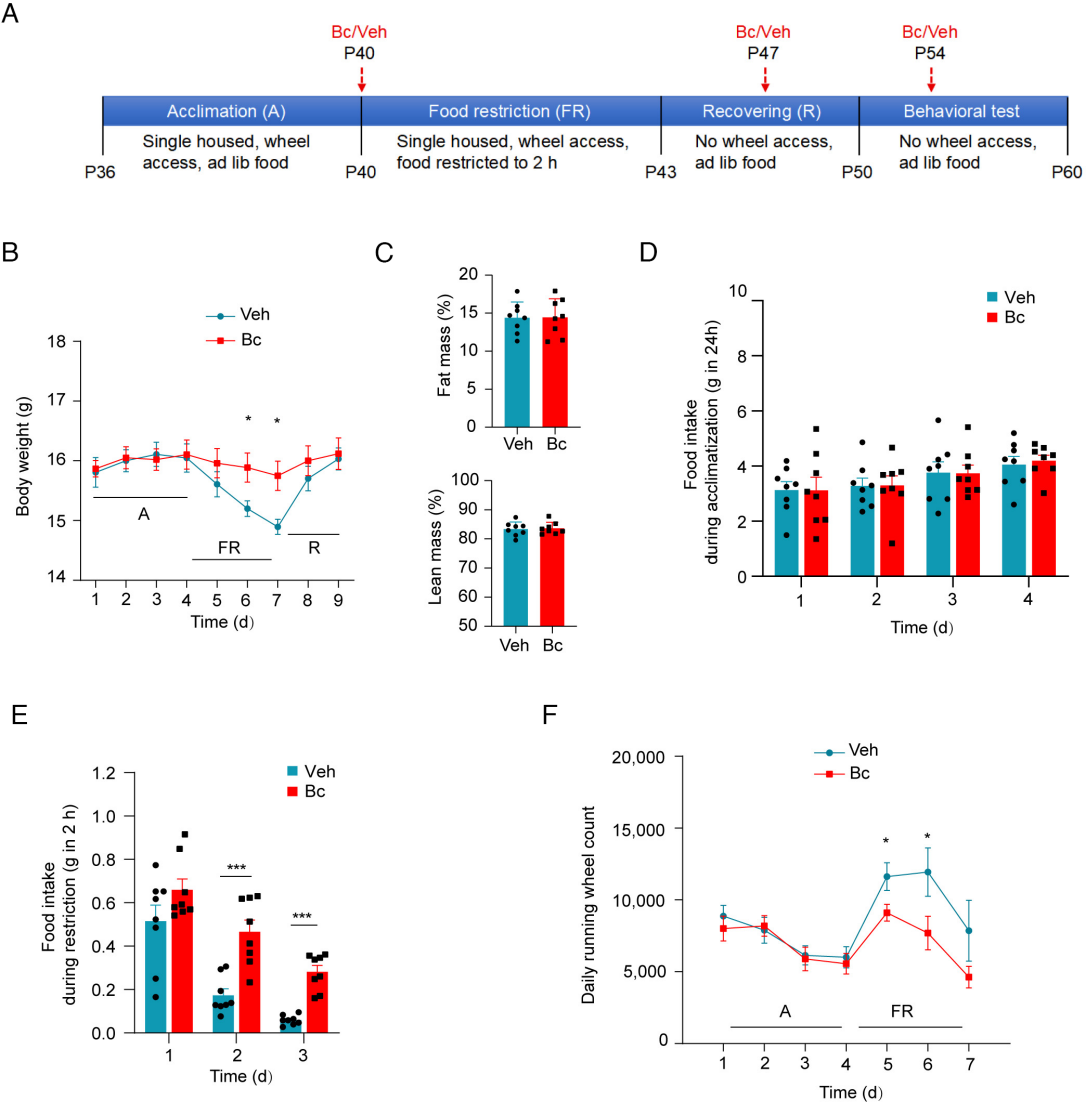
Bc attenuates anorexia. (*A*) Schematic diagram of experimental design. (*B*) Body weights of animals treated with vehicle or Bc during acclimation (A), food restriction (FR), and recovering period (R). (*C*) Body composition of mice measured during recovery (day 9) showing no difference between the groups. (*D*) Food intake during acclimation showing no difference between the groups. (*E*) Food intake during food restriction showing increased food intake in Bc-treated mice. (*F*) Daily running wheel activity during acclimation and food restriction. All data represent mean ± SEM. *n* = 8 mice per group. *P < 0.05 and ***P < 0.001 by two-tailed Student’s *t* test. (*C*–*E*) Each dot represents an animal.

AN is a life-threatening illness with poorly understood pathophysiological mechanisms. A number of animal models have been developed to study AN; the most widely used is the ABA model (2, 3). In this model, adolescent rodents are subjected to time-restricted feeding with unlimited access to a running wheel. The combination of these two factors leads to low caloric intake, significant weight loss, and excessive physical activity, recapitulating key features of the human condition (1, 3, 9). Using this paradigm, we have previously demonstrated that mice with AgRP neuron ablation and food restriction died within 72 h of compulsive running, whereas daily activation of AgRP neurons by way of chemogenetic tools prevented the mortality (9). The importance of AgRP neurons in AN was further underscored by a recent report showing that chemogenetic activation of AgRP neurons in mice during ABA attenuated body weight loss with reduction in excessive physical activity (25). Because treating mice with Bc activates AgRP neurons (Fig. 2) and induces hyperphagia (18), we tested whether Bc could be used to treat AN. Thus, peripubertal female mice were exposed to the ABA paradigm as previously described (9). Mice were singly housed at postnatal day 36 (P36) with ad libitum access to food, water, and a running wheel. After 4 d of acclimation at P40, mice were food restricted with free access to food for only 2 h daily for 3 d with free access to wheel running. At P40 and before the onset of food restriction, mice received the first i.p. injection of Bc or vehicle, with second and third injections performed at P47 and P54, respectively (Fig. 3*A*). There were no differences in body weight (Fig. 3*B*), daily food intake (Fig. 3*D*), and running wheel account (Fig. 3*F*) between Bc and control groups during acclimation. Similar to what was reported before (9), animals in the control group exhibited a progressive decline in body weight during food restriction; however, Bc-treated animals were able to maintain their body weights throughout the experiment (Fig. 3*B*). In addition, animals in the Bc group showed a significant increase in food intake during food restriction (Fig. 3*E*), with a parallel decline in compulsive wheel running (Fig. 3*F*). Given the hallmarks of AN being low caloric intake, progressive weight loss, and hyperactivity (1, 2), our results demonstrate that Bc is effective in mitigating AN by increasing food intake, preventing weight loss, and attenuating compulsive running.

### Bc Elicits Anxiolytic Effects.

A growing body of evidence has shown that exposing rodents in adolescence to the ABA paradigm activates the hypothalamic–pituitary–adrenal (HPA) axis and produces long-term anxiogenic effects in adulthood (2). For example, mice subjected to the ABA protocol displayed increased anxiety following the recovery period when their body weights had been restored (26). Importantly, AN patients show dysregulation of the HPA axis at both the neuroendocrine (increased corticotropic-releasing hormone) and the endocrine levels (increased cortisol) (27–29). We have reported that AgRP neuron–specific TET3 knockdown in mice reduces stress-like behaviors with decreased levels of circulating corticosterone (18). Given that Bc down-regulates TET3 protein in AgRP neurons (Fig. 2*A*), we subjected mice to behavioral tests after the recovery period (Fig. 3*A*). The open-field test (OFT) has been used to assess general locomotor activity and anxiety; the tail suspension test (TST) and forced swim test (FST) have been used to evaluate behavioral despair and also to test the efficacy of new antidepressant compounds. All three tests (OFT, TST, and FST) have been used in our previous studies to assess behavioral impacts of AgRP neurons in mice (9, 18).

**Fig. 4.**
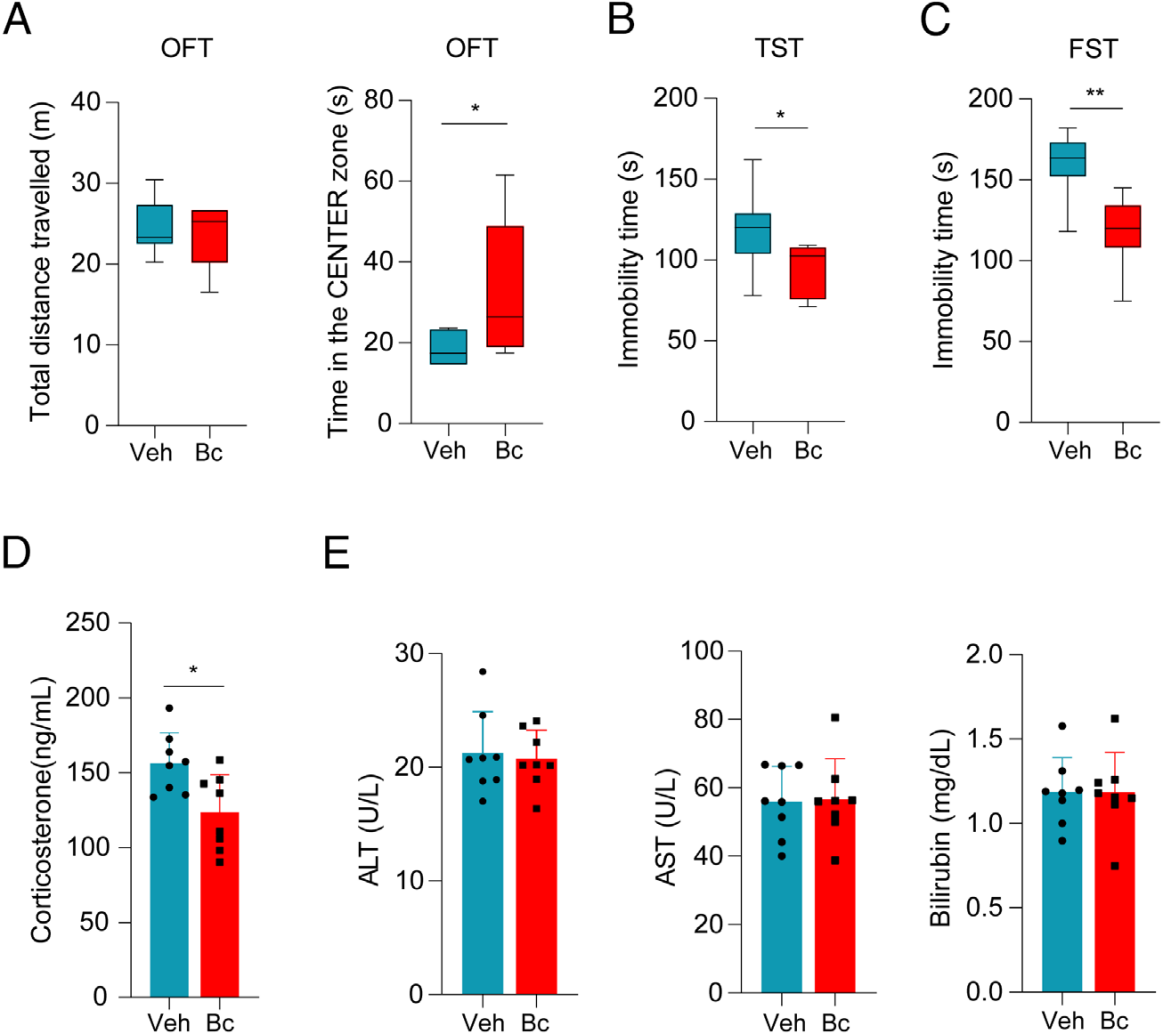
Bc elicits antistress effects. (*A*) Total distance traveled (*Left*) and time spent in the center zone (*Right*) in the open-field test. (*B*) Tail suspension test immobility scores. (*C*) Forced swim test immobility scores. (*D*) Plasma corticosterone concentrations. (*E*) Plasma ALT, AST, and bilirubin from mice showing no differences between the groups. All data represent mean ± SEM. *n* = 8 animals per group. *P < 0.05 and **P < 0.01 by two-tailed Student’s *t* test. (*D* and *E*) Each dot represents an animal.

During the recovery period, body weights of the animals were quickly restored within 3 d (Fig. 3*B*). No differences in body composition were observed between the groups at the end of the recovery period (Fig. 3*C*). In the OFT, we found no differences in the total distance traveled between Bc- and vehicle-treated groups (Fig. 4 *A*, *Left*), suggesting that Bc did not affect general locomotor activity. However, Bc-treated animals showed an increased time spent in the center zone (Fig. 4 *A*, *Right*), suggesting reduced anxiety. In addition, Bc-treated animals spent less immobility time than the controls both in TST (Fig. 4*B*) and FST (Fig. 4*C*), indicating decreases in depressive-like states. Furthermore, compared to control animals, the Bc-treated animals had reduced plasma cortisol levels (Fig. 4*D*). Finally, there was no evidence of liver toxicity following three weeks of once-a-week Bc injections (Fig. 4*E*). Taken together, our results show that Bc is effective in reducing anxiety/depressive-like states in mice.

## Discussion

Here, we use the activity-based paradigm shown to recapitulate characteristics of individuals with AN (1–3, 9, 25) and demonstrate the potential of Bc as a therapeutic for this disorder and associated anxiety/depressive behaviors. Specifically, we show that Bc acts to destabilize TET3 protein, although the exact mechanism remains to be determined. We also show that Bc phenocopies AgRP neuron–specific TET3 knockdown in that it activates AgRP neurons, simultaneously increases the expression of AGRP, NPY, and VGAT, and induces hyperphagia and anxiolytic effects. Thus, our results identify TET3 as a molecular, and AgRP neurons as a cellular, target of Bc action. However, we do not rule out potential effects of Bc on other cells/neuron circuits, either indirectly (via AgRP neurons) or directly. For example, AgRP neurons project to broad areas in the brain (5, 30); hence, Bc-induced changes in AgRP neurons could be relayed to other cells onto which AgRP neurons project. In addition, we have detected TET3 inhibition in non-AgRP cells in the ARC in Bc-exposed animals, although the effect appeared to be highly localized (*SI Appendix*, Fig. S1*B*). Through dose optimization, one may be able to minimize off-target effects of Bc, which warrants future investigation.

Notably, Bc is able to stimulate appetite via multiple routes of administration, including oral (18) and intraperitoneal (this work). Furthermore, Bc exerts its appetite-stimulating and antistress effects within 24 h, and its effects last for at least 1 wk following a single i.p. injection. This is in contrast to many current antidepressants which elicit antidepressant effects after 1 to 2 wk of taking the medication. Moreover, Bc appears to be well tolerated, and no liver toxicity has been detected after 3 wk of once-a-week i.p. injection.

Finally, as a proof of principle, our studies help to set the stage for more in-depth investigations including pharmacokinetic and toxicology studies before establishing Bc as a therapeutic for AN and perhaps cancer-induced anorexia and associated mood disorders.

## Materials and Methods

### Animals.

All procedures were approved by the Institutional Animal Care and Use Committee at the Yale University and were conducted accordingly. Female C57BL/6J mice (000664, Jackson Laboratories) were purchased. The *Agrp-IRES-Cre::LSL-Cas9-GFP* mice with GFP expression specifically in AgRP neurons were generated as previously described (18). Mice were housed at 22 °C to 24 °C with a 12-h light–12-h dark cycle with regular chow (Harlan Teklad no. 2018, 18% calories from fat) and water provided ad libitum. For all experiments, age-matched female animals were used. For experiments shown in Figs. 2–4, six to eight animals per group were used.

### Bc Treatment of Mice.

Bobcat339 powder (Sigma-Aldrich, SML2611) was freshly dissolved in dimethylsulfoxide (DMSO) at a concentration of 50 mg/mL and filtered through a 0.22-μm filter. It was further diluted with 1xPBS to a final concentration of 0.5 mg/mL before injections. Mice were injected i.p. with Bobcat339 at 1 mg/kg, 2.5 mg/kg, or 4 mg/kg.

### Cell Lines and Adenoviruses.

Mouse GT1-7 hypothalamic neuronal cell line (Sigma-Aldrich, SCC116) and human SH-SY5Y neuroblastoma cell line (Sigma Aldrich, 94030304) were purchased and cultured according to the manufacturers’ instructions. Purified Ad-TET3 adenovirus (Ad-FLAG.h-TET3, ADV-225322, Vector Biolabs) expressing human TET3 from a cytomegalovirus (CMV) promoter and Ad-GFP control adenovirus (1060, Vector Biolabs) were purchased.

### Cell Culture and Treatments.

For Bc treatment (Fig. 1 *A* and *B*), GT1-7 cells grown in 24-well plates at 2 × 10^5^ cells/well were incubated with vehicle or Bc at a final concentration of 10 μM for 6 h, followed by RNA and protein extractions. For TET3 protein stability assay (Fig. 1*B*), GT1-7 cells in 24-well plates at 2 × 10^5^ cells/well were incubated with vehicle or Bc at a final concentration of 10 μM for 3 h, followed by addition of CHX (Cell Signaling, 2112) at a final concentration of 50 μg/mL in the presence of 10 μM of Bc. Proteins were harvested at 0, 1, 2, and 3 h after addition of CHX. For TET3 expression restoration experiments (Fig. 1 *C* and *D*), GT1-7 or SH-SY5Y cells seeded in 24-well plates at 2 × 10^5^ cells/well were infected with Ad-GFP or Ad-TET3 at 4,000 gc/cell. Following 16 h of infection, vehicle or Bc was added at a final concentration of 10 μM. Protein and RNA were isolated 48 h later and analyzed.

### RNA Extraction and RT-qPCR.

Total RNAs were extracted from neuronal cells or homogenized hypothalamic arcuate nucleus tissue samples using the PureLink RNA Mini Kit (Ambion, 12183025). cDNA was synthesized using the PrimeScript RT Reagent Kit in a 20 μL reaction volume containing 0.5 to 1 μg of total RNA. Real-time quantitative PCR was performed in a 15 μL reaction volume containing 0.5 to 1 μL of cDNA using SsoAdvanced Universal SYBR Green Supermix in a Bio-Rad iCycler. Specificity was verified by melting curve analysis. The Ct values of each sample were used in the post-PCR data analysis. Gene expression levels were normalized against RPLP0. qPCR primers are listed in *SI Appendix*, Table S1.

### Western Blot Analysis.

GT1-7 and SH-SY5Y cells in 24-well plates were collected by manual scraping in 2× sodium dodecyl sulfate (SDS) sample buffer containing 1X phosphatase inhibitor cocktail (Thermo, 78427) and 1X protease inhibitor cocktail (Thermo, 78438), followed by heating at 100 °C for 5 min with occasional vortexing. The lysate was then centrifuged at 12,000 g for 5 min at room temperature (RT) to remove insoluble materials before loading onto 4 to 15% gradient SDS gels (Bio-Rad, 456-8086), followed by western blot analysis. The antibodies used were anti-TET3 (dilution 1:1,000; Active Motif, 61395), anti-TET2 (dilution 1:500; Cell Signaling Technology, 18950), and HRP-conjugated anti-GAPDH (dilution 1:5,000; Proteintech, HRP-60004). The secondary antibody was HRP-linked anti-rabbit IgG (dilution 1:10,000; Rockland, 611-1322).

### Immunofluorescence.

The immunofluorescence for brain slices was conducted using previous methods (18). In brief, postfixed sections were cut into 40-μm-thick sections, followed by five times washing. Then, the sections were incubated in a blocking solution for 20 min and incubated with anti-TET3 (dilution 1:2,000; Millipore Sigma, ABE290), anti-TET2 (dilution 1:500; Proteintech, 21207-1-AP), anti-AGRP (dilution 1:400; H-003-57, Phoenix Pharmaceuticals), anti-NPY (dilution 1:800, Cell Signaling Technology, 11976S), anti-VGAT (dilution 1:200; Abcam, Ab23592), or anti-FOS (dilution 1:1,000; Biosensis, R-1751-050) overnight at 4 °C. Negative controls were performed by omitting the respective primary antibodies. The next day, sections were washed five times and incubated in 0.4% Triton X-100 PBS with the secondary antibody donkey anti-rabbit IgG Fluor 594 (dilution 1:500; A-21207, Invitrogen) for 2 h at RT. The sections were coverslipped and scoped using a Keyence BZ-X700 fluorescence microscope. The fluorescence signals from GFP in AgRP neurons were detected without immunostaining.

### ABA Model.

On P36, animals were single housed with free access to food and water and 24 h access to a running wheel. After 4 d of acclimation, on P40, all food was removed from the cage and returned only for 2 h daily (free food access from 19:00 to 21:00) and 24 h access to a running wheel for 3 d. On P43, 24-h ad libitum access to food was returned, and the running wheel access was blocked to allow the animals to recover. The animals were allowed to recover for at least 1 wk before undergoing behavioral testing. Continuous multiday analysis of running wheel activity was recorded using Promethion Metabolic screening system (Sable Systems International). Food intake was measured after measurement of the food pellets before and after the 2-h food restriction. Body weight was monitored in the morning. Body composition was assessed using EchoMRI analysis.

### Behavioral Tests.

For all behavioral tests, mice were transferred to the testing room 1 h prior to testing for acclimation to the environment. All behavioral tests were performed in the afternoon (14:00 to 16:00). All behavioral apparatus were wiped with 70% ethanol prior to each trial and between trials. The open-field (OF) apparatus consisted of a 56- × 56-cm open arena with 30-cm-high walls. The mouse was placed into the center of the arena and allowed to move freely for 10 min with the activity being recorded and tracked by LimeLight 3 software (Actimetrics, Coulbourn Instruments). The software recorded and analyzed the distance and time traveled in the central (28- × 28-cm central area of the OF) and outer areas of the arena. The TST and the FST lasted for 6 min, and the total amount of immobility time during the final 4 min was measured for each animal.

### Blood Chemistry.

For corticosterone, blood samples were obtained via retro-orbital bleeding between 19:00 and 20:00. For alanine transaminase, aspartate transaminase, and bilirubin, blood samples were collected by cardiac puncture of terminally anesthetized animals. All blood samples were collected in EDTA tubes (Microtainer with K2EDTA, BD, 365974). The tubes were centrifuged at 2,000 × g at 4 °C for 20 min, and plasma was collected and stored at −80 °C until use. Plasma corticosterone levels were measured using the Corticosterone ELISA Kit (Enzo, ADI-900-097) according to the manufacturer’s instructions. Kits used to measure alanine transaminase (EALT-100) and aspartate transaminase (EASTR-100) were purchased from Bioassay Systems. The Bilirubin Assay Kit (MAK126) was purchased from Sigma Aldrich.

## Supplementary Material

Appendix 01 (PDF)

## Data Availability

All study data are included in the article and/or *SI Appendix*.
