# Supplementary material for: A small-molecule degrader of TET3 as treatment for anorexia nervosa in an animal model
Source: Proc Natl Acad Sci U S A. 2023 Apr 10;120(16):e2300015120. doi: 10.1073/pnas.2300015120 (PMC10120042; doi:10.1073/pnas.2300015120)
Supplement: Supplementary file 1 — Appendix 01 (PDF) [file pnas.2300015120.sapp.pdf]

Supplementary Information for

A small-molecule degrader of TET3 as treatment for anorexia nervosa in an animal model

Haining Lv[a,b], Jonatas Catarino[c], Da Li[a,d], Beibei Liu[a,d], Xiao-Bing Gao[c,e], Tamas L. Horvath[a,c,e,f], Yingqun Huang[a,e,1]

[1]Corresponding author.

Email: yingqun.huang@yale.edu

This PDF file includes:

Fig. S1

Fig. S2

Table S1

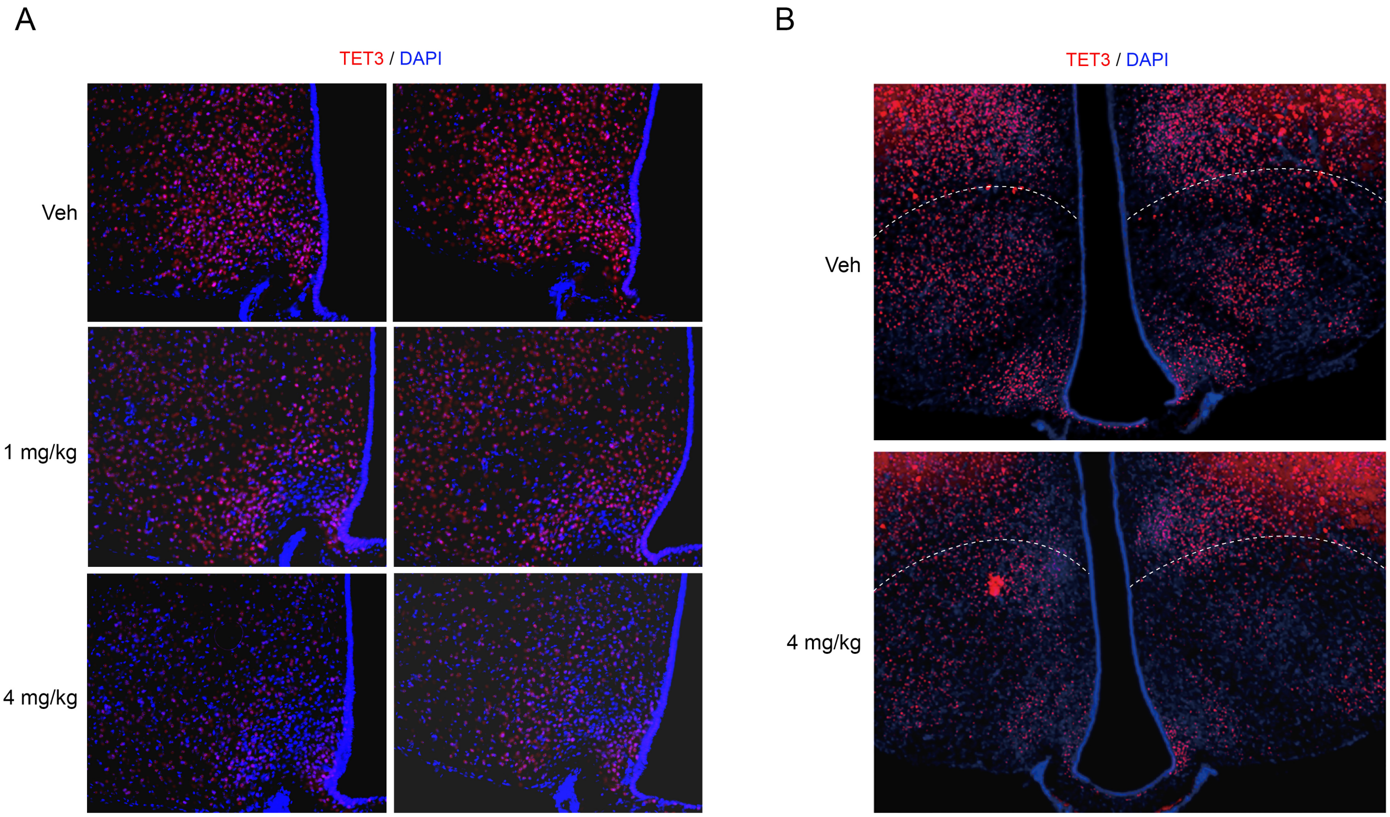

Fig. S1. Dose response of TET3 in the ARC. (*A*) Mice were i.p. injected with vehicle or Bc at 1 mg/kg or 4 mg/kg body weight. ARCs were isolated under fed conditions (9:00 – 11:00) 2 d later. Representative microphotographs of TET3 (red) are shown, with nuclei stained blue by DAPI. Each panel represents a mouse. (*B*) Mice were i.p. injected with vehicle or Bc at 4 mg/kg. ARCs were isolated under fed conditions (9:00 – 11:00) 2 d later. Representative microphotographs of TET3 (red) show localized Bc effects in the tip regions of the ARC.

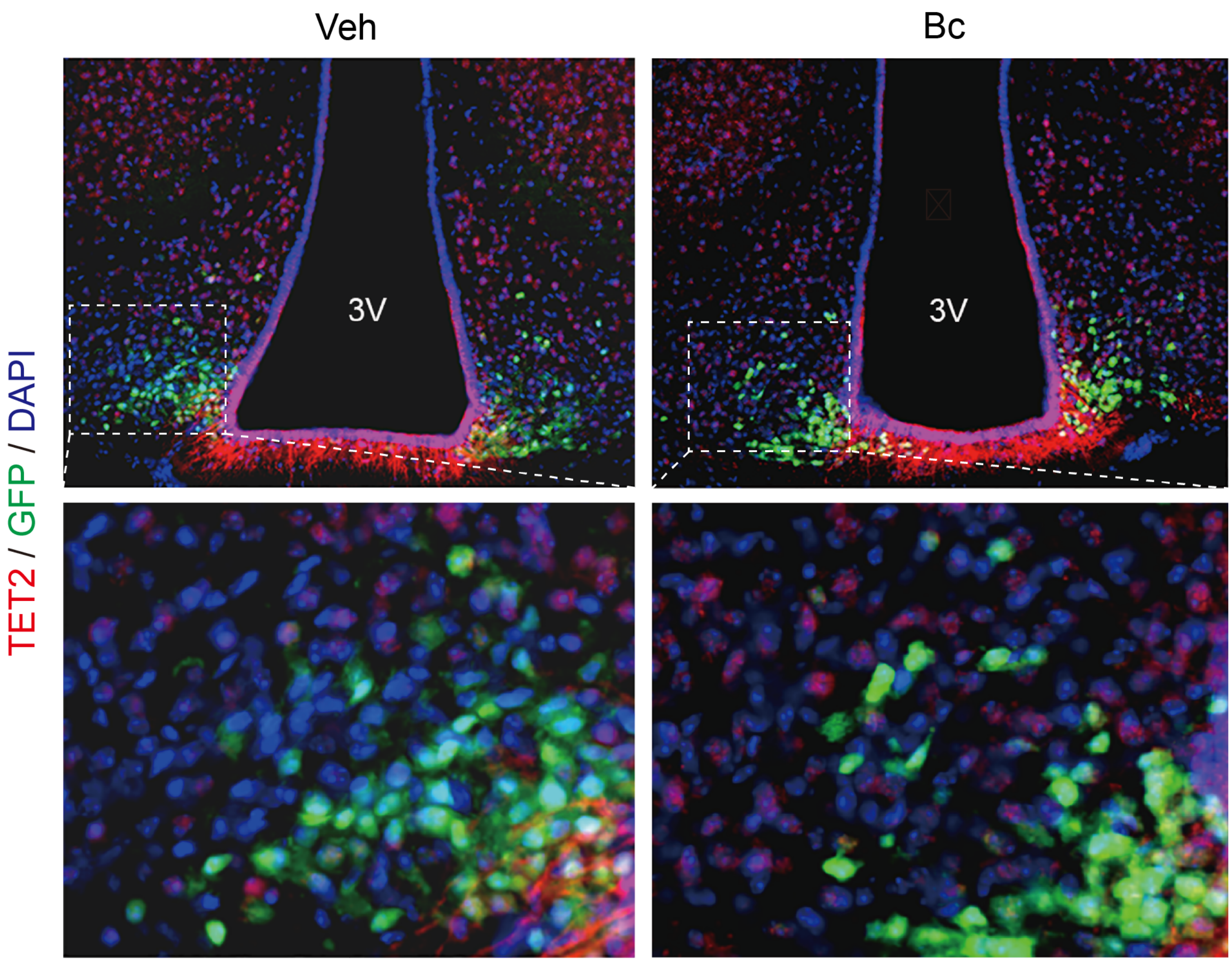

Fig. S2. TET2 expression in the ARC. Mice were i.p. injected with vehicle or Bc at 2.5 mg/kg. ARCs were isolated under fed conditions (9:00 – 11:00) 2 d later. Representative microphotographs of TET2 (red) and AgRP neurons (green) are shown, with nuclei stained blue by DAPI. Shown underneath are high magnifications of the outlined regions.

Table S1

**qPCR primer sequences**		
**Gene**	**Forward Primer**	**Reverse Primer**
Tet3 (mouse)	5'-CTTCCTATGGCTGGGAGTGAG-3'	5'-CTGCCTTGAATCTCCATGGTAC-3'
Agrp (mouse)	5'-GGCCTCAAGAAGACAACTGC-3'	5'-GCAAAAGGCATTGAAGAAGC-3'
Npy (mouse)	5’-AGGCTTGAAGACCCTTCCAT-3’	5’-ACAGGCAGACTGGTTTCAGG-3’
Slc32a1(mouse)	5’-TGGTCATCGCTTACTGTCTC-3’	5’-TGCTGCATGTTGCCTTCG-3’
Rplp0 (mouse)	5'-GATGGGCAACTGTACCTGACTG-3'	5'-CTGGGCTCCTCTTGGAATG-3'
TET3 (human)	5'-GACGAGAACATCGGCGGCGT-3'	5'-GTGGCAGCGGTTGGGCTTCT-3'
AGRP (human)	5'-GAAGAGGATCTGTTGCAGGA-3'	5'-CAGGACTCATGCAGCCTTAC-3
NPY (human)	5'-TCACCAGGCAGAGATATGGA-3'	5'-GCAAGTCTCATTTCCCATCA-3
SLC32A1(human)	Bio-Rad, qHsaCED0042869	Bio-Rad, qHsaCED0042869
RPLP0 (human)	5'-GGCGACCTGGAAGTCCAACT-3'	5'-CCATCAGCACCACAGCCTTC-3'
